# Supplementary material for: Assessment of computational methods for the analysis of single-cell ATAC-seq data
Source: Genome Biol. 2019 Nov 18;20:241. doi: 10.1186/s13059-019-1854-5 (PMC6859644; doi:10.1186/s13059-019-1854-5)
Supplement: Supplementary file 3 — Additional file 3: Review history. [file 13059_2019_1854_MOESM3_ESM.docx]

Review History

**First round of review**

**Reviewer 1**

**Are you able to assess all statistics in the manuscript, including the appropriateness of statistical tests used?**

yes

**Comments to author:**

This paper tried to address an current and important question in the analysis of single-cell ATAC-seq data. It collected 10 recent methods for pre-processing scATAC-seq data and evaluted their performance based on the clustering results on top of those pre-processing methods. The evaluation and comparison was based on 5 simulated datasets and 3 real datasets. The paper summarized the observations into some general recommendation on the choice of methods for scATAC-seq data processing. This paper involves a large amount of work, but the observations and conclusions obtained are vague, partially due to the nature of the complexity of the underlying biological question and imperfectness of data quality, and also partially due to questions in the current study. 
Examples of some major issues are:
1. The description of the method used for generating simulation data is quite blur. It is referred to the Method section in the main manuscript, but the Method section also only contains abstracted descriptions without any details. The 5 simulated datasets are actually only from two datasets by adding different levels of noise. The description in the Method section is so weak that readers cannot even find how noises were added to the data. There is no way to reproduce this work according to the description. 
2. Many of the compared methods are actually not directly comparable as they adopted very different approaches and the output "feature matrices" are not of the same type. It is not suitable to connect all of them to the same downstream clustering method and compare the results. Some methods have different application strategies and applicable scenarios. The authors claimed that the framework for comparing methods is "unbiased" but didn't try to provide any data or discussion to support this claim, as it is not clear how representative the selected simulation data and real data are. 
3. Even if the comparison were fair, the conclusion is very vague. The observations of comparisons on different datasets have noticeable differences. It is not clear how convincing the observations are based on the datasets tested in this work are. The provided data cannot fully support drawing the conclusion and recommendations.
4. Even if the conclusions were solid, the underlying reason of why the methods perform differently should be analyzed from either computational view or biological view, or both.
Discussion:
Another question that puzzles the reviewer is about the information content of current scATAC-seq data. The paper introduced in the Background that "only 1-10% of expected peaks are detected in single cells from scATAC-seq data". It is known that the current efficiency of capturing open regions in single cell genomes is low, but the paper didn't provide any source for this range. More importantly, this low proportion raises the question that how much information about cell types or cell states can be captured in current scATAC-seq data. The successful results provided in this paper and other papers show that some major cell types can be well clustered with scATAC-seq data. Does this mean that <10% of all expected peaks are sufficient for the analysis? If yes, how this 10%? Why scATAC-seq can happen to capture the most important peaks? The situation in scRNA-seq data is different. Although scRNA-seq data also have low efficiency in capturing all expressed genes (20-50% according to this paper), one can reasonably believe that those highly expressed genes have a higher probability of being captured. Although scRNA-seq misses many signals, it captures the strongest signal and therefore can discriminate major cell type or cell state information with the incomplete information. But chromatin openness is binary in nature, and so there is no reason why the randomly captured 1-10% signal can reflect the information we want. Discussing this question may be out of the scope of the current paper. But without an understanding on this question, it is hard to understand why some ways of building the feature matrix work and some other ways may not work.

**Reviewer 2**

**Are you able to assess all statistics in the manuscript, including the appropriateness of statistical tests used?**

yes

**Comments to author:**

Chen and colleagues perform an assessment of methods for the processing and analysis of scATAC-seq data. They use both simulated and real datasets to systematically ascertain which approaches tend to consistently work better in across different settings. Overall, this is a challenging task as there are vast numbers of ways to do the simulations, fine-tune the parameters for each approach, and evaluate the results. Overall, the authors have been comprehensive and have tackled this task with rigor and transparency in their choices. They have found that several approaches should clearly not be used for de novo clustering and several other algorithms perform relatively well. The ability of snapATAC to rapidly process and analyze large data is also an important finding. Furthermore, they compare the pseudo-bulk vs. clade-bulk strategies. Overall, this benchmarking paper will be of interest to the single cell community and serve as a valuable guide for future methods developers.

Major comments:

1) One analysis that was not performed but would be of interest to researchers in this field is how does sequencing depth affect results. One question always asked is "Will deeper sequencing give new or better-defined clusters?". Although the effect of noise was assessed, it appears as though the same "depth" was used in all simulations (i.e. 1,000 fragments). What happens if this number is decreased? Do some approaches perform better or worse when the data is sparser? 

2) Some of the methodology for simulation was unclear. For example, the statement "For a simulated single cell, we generate a vector of per-peak probabilities weighted by the bulk intensity at each peak and the number of fragments in peaks desired, and simulate the observation of a fragment in each peak using the R rbinom function (size = 1)." Could a more formal definition of "weighted by the bulk intensity at each peak and the number of fragments in peaks desired" be given?  

3) Similar to above, it was not fully clear in the methods how noise was being added. Could the sentence "To introduce noise, per-peak rates were adjusted by adding a fixed term that was subsequently rescaled" be expanded? Formulas may be helpful as well.  

4) SnapATAC excludes certain regions that were blacklisted by Encode. Do other algorithms do this at all? Does this step greatly contribute to its increased accuracy or are their other steps that can be attributed to its top rank? 

Minor comments: 

1) In the text the term "naïve" is mostly used for the "control-naïve" dataset. However, in the figures, the term "control" is used more frequently. It would be clearer to use the exact same terminology throughout.

2) For the speed performance comparison, were all methods run in parallel with the full 44 cores? This would make a large difference in performance if some methods were parallelizable and others were not.

3) Some casual language is used throughout including contractions at lines 293, 319, and 381 ("didn't" and "don't").

4) This is a grammar mistake in Supp Fig 1 legend. The word "of" is repeated. 

5) For the organization of the "noise" related supplementary figures, it would be easier for the reader to digest if all results/umaps for different noises and clustering algorithms were put in a single plot and then have separate figures for each pipeline/approach (i.e. a single figure containing all of snapATAC noise results). Then one can more readily ascertain the effect of noise on each approach. Currently, it is hard to see the effect of noise on a single approach when having to flip back and forth between figures.

**Authors Response**

**Point-by-point responses to the reviewers’ comments:**

Comment

*Response*

Reviewer reports:

Reviewer #1: This paper tried to address a current and important question in the analysis of single-cell ATAC-seq data. It collected 10 recent methods for pre-processing scATAC-seq data and evaluated their performance based on the clustering results on top of those pre-processing methods. The evaluation and comparison was based on 5 simulated datasets and 3 real datasets. The paper summarized the observations into some general recommendation on the choice of methods for scATAC-seq data processing. This paper involves a large amount of work, but the observations and conclusions obtained are vague, partially due to the nature of the complexity of the underlying biological question and imperfectness of data quality, and also partially due to questions in the current study.

*We agree completely with the reviewer’s concern regarding the vague discussion and summary of our findings. Therefore, we have rewritten the Discussion and Conclusions sections to better highlight the key findings of our study and its limitations. We intend for this manuscript to be a complete, concise, and reader-friendly reference such that a non-expert could make use of our conclusions just as well as a researcher who might be interested in designing new methods to address some of the outstanding challenges presented by the analysis of scATAC-seq data.*

*Additionally, we appreciate the reviewer’s recognition of the complexity of the task at hand; a fair assessment of the available methods is a challenging task; in fact, there are several considerations including but not limited to:*

*metrics, computational requirements and available assays. Although there are many underlying biological questions that can be explored with scATAC-seq assays, we focused here on the task of discovering homogenous subpopulations of cells that are similar by type or state, by unsupervised clustering.*

Examples of some major issues are:

1. The description of the method used for generating simulation data is quite blur. It is referred to the Method section in the main manuscript, but the Method section also only contains abstracted descriptions without any details. The 5 simulated datasets are actually only from two datasets by adding different levels of noise. The description in the Method section is so weak that readers cannot even find how noises were added to the data.

There is no way to reproduce this work according to the description.

*We thank the reviewer for raising this important point. We acknowledge that our simulation method was not properly documented. To address this problem, we have:*

*1) Extended the main text and the Methods section, adding more details, which illustrate the motivation behind our procedure. This includes adding specific steps and parameter choices. The updated Methods section explains in detail the nature of the noise, based on the provided equations. Briefly, the noise level is a way to control the informativeness of each peak/region. If a region is shared across all of the cell types with the same intensity, this region is not informative in discriminating subpopulations, whereas highly cell type-specific peaks with skewed intensity in one or few populations are quite useful in separating populations. In other words, the noise level acts to control the specificity of each peak. A noise level of 0 preserves the original specificity, while a noise level of 1 makes it equally likely to observe fragments from a peak regardless of the cell type, thus destroying its information content and making the discrimination of the different populations impossible.*

*Main text:*

*“Briefly, to generate the peak by cell matrices, we defined a noise parameter (between 0 and 1) as the proportion of reads occurring in a random peak from one of the sorted populations. The remaining proportion of reads was distributed as a function of the bulk sample (see Methods). A feature matrix with a noise level of 0 preserved perfectly the underlying cell type specificity of the reads within peaks.*

*Conversely, a feature matrix with a noise level of 1, contained no information to discriminate cell types based on the reads within peaks. In our study, we considered three noise levels: no noise (0), moderate noise (0.2) and high noise (0.4).”*

*Methods section:*

*“In order to evaluate and benchmark various approaches, we generated synthetic (labeled) data from down-sampling 18 FACS-sorted bulk populations that were previously described [28]. For ease of interpretation, we considered only 6 isolated populations (HSC, CMP, NK, CD4, CD8, Erythroblast). For the erythropoiesis simulation, eight additional populations (P1-P8) originally described in [18] were also considered.*

*Our simulation framework starts with a peak x cell type counts matrix (from bulk ATAC-seq) and generates a single-cell counts matrix (𝐶) for an arbitrary number of synthetic single cells. Explicitly, for a simulated single cell 𝑗 and corresponding peak 𝑖 from bulk cell type 𝑡, we seek to generate 𝑐&,( where 𝑐&,( ∈ {0,1,2}, noting that these values correspond to possible observations in a diploid genome. Next, we define the rate (𝑟& 1) at which the peak 𝑖 is prevalent in the bulk ATAC-seq data for cell type 𝑡. This rate is determined by the ratio of reads observed in peak 𝑖 over the sum of all reads. Assuming a total of 𝑘 peaks for the matrix 𝐶 and for user-defined parameters 𝑞 (noise parameter; 𝑞 ∈ [0,1]) and 𝑛 (number of simulated fragments), we define 𝑐&,( as follows:*

*Intuitively, the parameter 𝑝&1 defines the probability that a count will be observed in peak 𝑖 for a single cell. Additionally, 𝑝&1 can be decomposed into the sum two terms. As 𝑞 → 0, the first term dominates, and the probability of observing a count in peak 𝑖 is simply the scaled probability of the ratio of reads for that peak from the bulk ATAC-seq data (𝑟&1). Thus, when 𝑞 = 0, the simulated data has no noise. Conversely as 𝑞 →1, the second term dominates, and 𝑝&1 reduces to a flat probability that is no longer parameterized by the peak 𝑖 or cell type 𝑡 and thus represents a random distribution of 𝑛 fragments into 𝑘 peaks.*

*For all simulations, we simulated 200 cells per labeled cell type. In the base simulations, we parametrized 𝑛 = 1,000 fragments in peaks in expectation for all cells. For additional simulations that compared different data coverages, we set 𝑛 to various values to benchmark this effect. To evaluate the effect of noise in our simulation, we generated data with 𝑞 = 0, 0.2 and 0.4. At values of 𝑞 > 0.4, no method could reliably separate the simulated cells populations. Finally, since our simulation started at the reads in peaks level, for some methods, the core algorithm associated with the method was extracted in order to benchmark it in this setting. Additionally, full code to reproduce these simulated dataset matrices has been made available with our online code resources.”*

*2) To ensure the reproducibility of our simulation and further clarify the procedure by which it was performed, we have provided all scripts used to generate synthetic datasets. In addition, we now provide all scripts needed to reproduce the entire benchmark analysis on GitHub and in the form of supplementary data.*

*“Importantly, we provide more than 100 well-documented Jupyter Notebooks (https://github.com/pinellolab/scATAC-benchmarking/) to easily reproduce our analyses. We anticipate that this will be a valuable resource for future scATAC-seq benchmark studies.”*

*3) Moreover, to assess the robustness of the different methods and their ability to distinguish cell populations despite sub-optimal sequencing depth, we have generated 5 new datasets with various read coverages in addition to the five synthetic datasets with different noise levels considered in the previous version of this manuscript (see response to Reviewer #2, point 1).*

2. Many of the compared methods are actually not directly comparable as they adopted very different approaches and the output "feature matrices" are not of the same type. It is not suitable to connect all of them to the same downstream clustering method and compare the results. Some methods have different application strategies and applicable scenarios. The authors claimed that the framework for comparing methods is "unbiased" but didn't try to provide any data or discussion to support this claim, as it is not clear how representative the selected simulation data and real data are.

*Thank you for raising this important point; we agree that our scope was not entirely clear in the previous version of this manuscript and that our assessment of the different methods appeared not well-justified. When designing this study, we reasoned that a benchmark procedure could be approached from two very different perspectives.*

*The first is the end user perspective, that of a user that runs a method as a black box, following the provided documentation with the goal of obtaining a reasonable clustering solution without worrying too much about internal design choices or procedures. In such a setting, we agree that it is hard to compare the methods as they may use different transformations, featurization, and clustering approaches (if at all). With this benchmarking design, it is not trivial to assess the value of each step and especially, the proposed featurization by each method.*

*The second perspective is that of the developer or someone who is interested in developing new methods for scATAC-seq clustering and would like to learn what are the best features, transformations, and modeling steps that might influence a common clustering procedure. In our framework, even if the resulting feature matrices (that represent the core of each method) are obtained using different approaches, the downstream steps adopted for benchmarking the methods are fixed. This fixed downstream evaluation uses clustering algorithms, which are well-accepted by the single cell field (see Kiselev, Andrew and Hemberg, Nat. Rev. Genet., 2019) as well as classic clustering evaluation metrics (ARI, AMI, Homogeneity). It is worth pointing out that even if the feature matrices are of different nature and dimensions, each clustering solution assigns a discrete label to each cell and the solutions are a common ground on which we have based our comparisons. This strategy provides a fair assessment guided by common clustering metrics by which we can compare the value of each featurization method on a given clustering approach.*

*In the revised version of the manuscript, we reasoned that it is important to spell out these two perspectives and also provide additional insights on the end user perspective since some readers will use the methods as end to end pipelines. Therefore, we have completed our above-proposed vision with some additional analyses. As the reviewer has pointed out, different methods have different application strategies and scenarios in which they might be useful, therefore we restricted these additional analyses to methods that propose a clustering strategy in their documentation.*

*“Clustering performance when running methods as end to end pipelines When designing this study, we reasoned that a benchmark procedure could be approached from two very different perspectives. The first is the end user perspective, i.e. a user that runs a method as a black box following the provided documentation with the goal to obtain a reasonable clustering solution without worrying too much about the internal design choices and procedures. In these settings, it is not trivial to systematically compare the methods and understand which part related to the featurization may influence the final clustering performance, especially if also the clustering algorithms used are different. The second perspective that was used instead in the rest of this benchmarking effort is the developer perspective, i.e. we tried to understand what are the key steps of each method that can boost clustering performance of common clustering approaches. Regardless, we reasoned that it is important to provide some insights on the user perspective, since some readers will use the tested methods as end-to-end pipelines. Therefore, we also compared the clustering solutions produced by running the complete analysis pipelines as outlined in tutorials for the methods that explicitly implement a clustering step (see Sup Note 6). We evaluated the clustering results using ARI, AMI and Homogeneity for the Buenrostro2018 and sciATAC- seq mouse datasets, and RAGI for the PBMCs 10X dataset (Sup Table 16-17-18). We observe the top three methods, i.e. Cusanovich2018, cisTopic and SnapATAC, still outperform the other methods but with a slightly different ranking. (Cusanovich2018 is ranked first followed by cisTopic and SnapATAC, Fig. 7b, Sup Fig. 13b). Also, both scABC and Cicero performed better than Scasat in this analysis. Interestingly, we observed that SnapATAC, cisTopic, Cusanovich2018, and Scasat have even better clustering solutions in our benchmarking framework compared to using their own clustering approach. On the other hand, scABC and Cicero had better clustering results when running their own clustering procedure. scABC uses an unsupervised clustering method tailored to single cell epigenomic data (including scATAC-seq). Although it uses the naïve peaks-by-cells raw count as its feature matrix, it calculates cells weights by considering their sequencing coverage and giving more weight to cells with higher number of reads. Also, it performs two steps of clustering by using weighted k-medoid algorithm based on Spearman rank correlation to find landmarks first and then assigns cells to the landmarks. These specific steps help improve its clustering performance. For the Cicero clustering workflow, we used the gene activity scores and, as proposed in their tutorial, functions from Monocle2, to (i) normalize the scores and (ii) reduce the dimensionality with tSNE by using the top PCs before clustering cells. These extra steps helped in improving its clustering solutions.*

*This suggests that appropriate normalization steps need to be properly performed to improve clustering analysis, in addition to simple transformations like binarizing counts and/or performing a PCA.”*

*Interestingly, we learnt that some combinations of feature matrices with the simple clustering approaches we employed perform even better than the original combination proposed by the respective authors. We believe this highlights the value of such a dual-characterization of both featurization and clustering methods. Further, we hope the reader will be able to make a more informed choice of analysis pipeline based on the summary we have provided here.*

*Regarding the choice of datasets analyzed, we attempted to create a simulation that recapitulates our current understanding of peaks obtained from the profiling of chromatin accessibility by the Tn5 transposase. We acknowledge that a simulation framework will never completely recapitulate reality, therefore we have added a few statements to our discussion to highlight such limitations and the required assumptions that were made. To improve insight gained from the real datasets, we focused on available assays based on complementary technologies and for which we had access to high-quality published data (the 10X microfluidics commercial platform that is droplet based, the sci-ATAC protocol which uses combinatorial indexing through split-pooling, and the accurate but low throughput array-based protocol used in Buenrostro et. al., 2018). We believe this approach provides a balanced exploration of the performance of various methods in highly divergent scenarios.*

*“We are aware of current limitations in our benchmarking effort. We have compared single cell ATAC-seq methods based on their ability to separate discrete cell populations; however, this might not be ideal when dealing with a continuous cell lineage landscape. We observe that chromVAR generally works better in preserving a continuous space while SnapATAC tends to break a putative landscape into discrete populations. The choice of method is ultimately case-specific and may be driven by the downstream application. For example, the feature matrix obtained by chromVAR may be more suitable for trajectory inference [26] while the one obtained from SnapATAC may be more appropriate better identify discrete and well separated cell populations by clustering. We acknowledge also that not all tested methods were specifically designed to produce clustering results. For example, chromVAR, Cicero, and Gene Scoring were designed to determine important marker genes, their regulatory logic, or to infer enriched TF binding sites within accessible chromatin regions. However, because clustering is a critical part of single-cell analysis and researchers frequently use output from all methods to produce clustering results [1], we felt that evaluating the clustering abilities using feature matrices produced by each method was a useful measure. An additional limitation of our study is that it is impossible to create a simulation framework that models an experimental outcome with perfect accuracy. Several assumptions were made to enable our simulation of the data; these assumptions are described in the methods section of this manuscript, where we detail explicitly how the simulated data was generated.*

*Interestingly, we learnt that some combinations of feature matrices with the simple clustering approaches included in our benchmarking framework perform even better than the original combination proposed by the respective authors. This highlights the value of this dual-characterization (user vs designer perspective) and provides a summary of both perspectives to the readers.”*

3. Even if the comparison were fair, the conclusion is very vague. The observations of comparisons on different datasets have noticeable differences. It is not clear how convincing the observations are based on the datasets tested in this work are. The provided data cannot fully support drawing the conclusion and recommendations.

*We appreciate the reviewer’s insightful comment and therefore we have rewritten the Discussion and Conclusion sections to reflect better the key points of our benchmarking effort and the two perspectives illustrated in the previous point.*

*We believe the key points of our study are:*

*1) A few methods consistently outperform the other methods regardless of the dataset used, and some methods are worse than a naïve approach based on a simple PCA on the peak x cell matrix. We have also expanded the discussion on computational requirements with a focus on running times. An overall ranking across datasets that clearly illustrates these points is now summarized in Figure 7 and in the Conclusions.*

*“Our benchmarking results highlight SnapATAC, cisTopic, and Cusanovich2018 as the top performing scATAC-seq data analysis methods to perform clustering across all datasets and different metrics. Methods that preserve information at the peak-level (cisTopic, Cusanovich2018, Scasat) or bin-level (SnapATAC) generally outperform those that summarize accessible chromatin regions at the motif/k-mer level (chromVAR, BROCKMAN, SCRAT) or over the gene-body (Cicero, Gene Scoring). In addition, methods that implement a dimensionality reduction step (BROCKMAN, cisTopic, Cusanovich2018, Scasat, SnapATAC) generally show advantages over the other methods without this important step. SnapATAC is the most scalable method; it was the only method capable of processing more than 80,000 cells. Cusanovich2018 is the method that best balances analysis performance and running time.”*

*2) Choice of pseudo-bulk vs bulk peaks does not significantly affect clustering performance.*

*“Bulk ATAC-seq peaks vs aggregated scATAC-seq peaks*

*To evaluate the effect of using peaks obtained from bulk ATAC-seq data versus peaks obtained from aggregated single cell profiles, we reanalyzed the Buenrostro2018 dataset in which both are available (Sup Fig. 15-16). Here we considered only the methods that use peaks as input (i.e. SnapATAC, SCRAT, BROCKMAN are excluded). For the aggregated scATAC-seq peaks, we merged cells of the same cell type based on the FACS sorting labels and performed peak calling within each cell type. Then peaks defined within each cell type were merged. For most methods we did not observe clear differences in performance between the two input peak strategies. For cisTopic, Cusanovich2018, and Cicero, aggregated scATAC-seq*

*peaks overall perform better across all three metrics (Sup Fig. 17a, Sup Table 19).*

*We also tested the strategy of defining pseudo bulk samples from clades when no sorting labels are provided. Cusanovich2018 is the only method that provides a workflow to identify initial clades and call peaks within each clade. It counts reads within the fixed-size windows and pre-clusters cells using hierarchical clustering to define initial clades from which peaks are called. We applied this strategy to all three real datasets (Sup Fig. 18). We observed that in all three datasets, Cusanovich2018 performs well in identifying the isolated major groups and the identified clades match well the labels provided, including FACS-sorted labels, cell-ranger clustering solutions, and known tissues labels. Overall the Cusanovich2018 ‘pseudo bulk’ strategy for defining de novo peaks is able to capture the heterogeneity within single cell populations and can serve as a promising unsupervised way to define pseudo bulk subpopulations and to perform peak calling.”*

*3) With the exception of SnapATAC, current computational methods were not designed such that they could handle large datasets. We did an in-depth analysis of their source code to highlight current implementation choices that can be problematic and suggest some available solutions (Sup Note 6). We also provide now the running time for the different methods to highlight another important aspect of their scalability (Sup Figure 22, Sup Table 20). Finally, we also compared running time vs performance to suggest which method could offer a good clustering solution in a reasonable time (Figure 7C).*

*“Main text:*

*SnapATAC is the most scalable method; it was the only method capable of processing more than 80,000 cells. Cusanovich2018 is the method that best balances analysis performance and running time.*

*Sup Note 5:*

*As mentioned in the main text, SnapATAC is the only methods that allows to process successfully large datasets, as the sciATAC-seq mouse dataset with ~80000 cells. Here we investigate why the other methods failed to analyze this large dataset. We hypothesize that main reason is related to the way the methods*

*load/process the data in memory. In fact, we discovered that several methods require to load the entire dataset in the central memory (RAM).*

*BROCKMAN, Cicero and Gene Scoring try to load the entire dataset in memory using the read.table function or the fread function within the data.table package in R. Other methods such as: Cusanovic, Scrat, chromVAR, scABC and Scasat, store the entire dataset in memory within a Matrix object in R. CisTopic, has an optimized step to map the reads into the genome using the Rsubread function. This function creates a hash table of the entire genome and allows the user to select the amount of memory to use. At the end, the entire dataset is stored in the computer memory in a CisTopicObject data structure.*

*SnapATAC, preprocess the entire dataset and store it a .snap file. This file is based on the HDF5 technology that allows out of core computation. In SnapATAC the Python library h5py (a wrapper for HDF5 core library) is used to create the custom snap file format. More information about this custom file are available here : https://github.com/r3fang/SnapTools/blob/master/docs/snap_format.docx .”*

*4) As we mentioned in the previous point, a dual-perspective analysis not only allows one to decouple the featurization and the clustering procedure but also to explore the performance of novel combinations as compared to those proposed by the respective authors. These novel combinations might serve as starting points for the development of novel methods.*

4. Even if the conclusions were solid, the underlying reason of why the methods perform differently should be analyzed from either computational view or biological view, or both.

*As discussed in prior points, we have heavily updated the analysis of the source code as well as the computational components of each method that distinguishes their performance from one another.*

Discussion: Another question that puzzles the reviewer is about the information content of current scATAC-seq data. The paper introduced in the Background that "only 1-10% of expected peaks are detected in single cells from scATAC-seq data". It is known that the current efficiency of capturing open regions in single cell genomes is low, but the paper didn't provide any source for this range. More importantly, this low proportion raises the question that how much information about cell types or cell states can be captured in current scATAC-seq data. The successful results provided in this paper and other papers show that some major cell types can be well clustered with scATAC-seq data. Does this mean that <10% of all expected peaks are sufficient for the analysis? If yes, how this 10%? Why scATAC-seq can happen to capture the most important peaks? The situation in scRNA-seq data is different. Although scRNA-seq data also have low efficiency in capturing all expressed genes (20-50% according to this paper), one can reasonably believe that those highly expressed genes have a higher probability of being captured. Although scRNA-seq misses many signals, it captures the strongest signal and therefore can discriminate major cell type or cell state information with the incomplete information. But chromatin openness is binary in nature, and so there is no reason why the randomly captured 1-10% signal can reflect the information we want.

Discussing this question may be out of the scope of the current paper. But without an understanding on this question, it is hard to understand why some ways of building the feature matrix work and some other ways may not work.

*This is another important consideration. We believe the distinction between biological and computational performance is important, especially in the context of unsupervised clustering. A big and critical assumption (or hope) of our field is that an unsupervised clustering procedure will provide clustering solutions that recapitulate different populations corresponding with different cell types/states. Given that for several real datasets the ground truth is not known, a current compromise during the exploratory clustering analysis is to use known marker genes, sorted populations or time points to validate the clustering solutions based on classic metrics. If we embrace this assumption, keeping in mind that additional validation is required to truly delineate the subpopulation structure of a population of cells, the two views, biological and computational can be reconciled.*

*Our benchmark procedure is aimed to provide some guidelines based on explorative analyses that are currently adopted in several published papers.We updated the discussion to reflect this point.*

*“We believe it is important to stress the distinction between biological realities and computational performance, especially in the context of unsupervised clustering. A big and critical assumption (or hope) of our field is that an unsupervised clustering procedure will provide clustering solutions that recapitulate different populations corresponding to different cell types/states. Given that for several real datasets the ground truth is not known, a current compromise during the exploratory clustering analysis is to use known marker genes, sorted populations or known tissues to validate the clustering solutions based on classic metrics. If we embrace this assumption, keeping in mind that additional validation is required to truly delineate the subpopulation structure of a population of cells, the two views, biological and computational can be reconciled. Our benchmark procedure is aimed to provide some guidelines based on explorative analyses that are currently adopted in several published papers.”*

*We also thank the reviewer for the thoughtful point on the relative informativeness of peaks with respect to scATAC-seq vs that of scRNA-seq. We agree that discussing the raised question in general is out of the scope of the current paper. However, we performed two simple analyses to provide some guidelines on how many informative peaks may be necessary for clustering subpopulations. Briefly we downsampled bulk reference peaks for one real dataset and one synthetic dataset using two methods that explicitly perform a peak selection procedure. We observed reasonable and stable clustering performance using more than 20% of the ranked peaks.*

*As the number of peaks is reduced, the scores start to decline noticeably and decrease almost monotonically. Below 1%, both methods perform poorly. However, this analysis cannot be generalized, since as we have shown in the section “Rare cell type-specific peak detection” it is also important to include in this reasoning the subpopulation structure of the sampled population and in particular the frequency of rare populations. Finally, we have described now the effect of removing the first principal component, which contributes to answering the question raised regarding capturing signal in scATAC to discriminate cell types.*

*“To evaluate the effect of selecting peaks based on their representation in the cell population or based on their intensity (defined as the sum of reads in that peak in all samples), we focus on the two methods that implement the step of peak selection, Cusanovich2018 and Control-Naïve (equivalent to scABC+PCA).*

*To assess the two peak selection strategies, we ran both Cusanovich2018 and Control-Naïve on both simulated bone marrow dataset at noise level of 0.2 with a coverage of 2500 fragments and the Buenrostro2018 dataset by varying the cutoffs for peak inclusion (Sup Fig. 20-21). We calculated the intensity of peaks by counting the number of reads across all cells and calculated the frequency of peaks by counting the number of cells in which a peak is observed. For this analysis we selected the top peaks based on intensity and frequency with the following cutoffs: top 100%, 80%, 60%, 40%, 20%, 10%, 8%, 6%, 4%, %2, 1%.*

*For both Cusanovich2018 and Control-Naïve, the two peak selection strategies have similar clustering result scores when varying the cutoff (Sup Fig. 20a-b,21a-b). We observed reasonable and stable clustering performance using more than 20% of the ranked peaks. As the number of peaks is reduced, the scores start to decline noticeably and decrease almost monotonically. Below 1%, both methods perform poorly. Keeping the first PC vs removing the first PC In preparing this manuscript, we noticed that in some cases, the first principal component (PC) may only capture variation in sequencing depth instead of biologically meaningful variability. To make a thorough assessment of how the first PC affects the clustering results, we compared the effect of keeping vs removing the first PC on the three real datasets (for this comparison we consider both the methods that implemented PCA and the combination of PCA and the methods that did not implement a dimensionality reduction step) (Sup Fig.14). Across all three datasets, we observe that for Control-Naïve, BROCKMAN, SCRAT-PCA, and Gene Scoring-PCA, removing the first PC consistently helped in better separating the different populations in UMAP projections and improved clustering performance. In contrast, the performance of chromVAR-PCA with motifs as features consistently dropped after removing the first PC. Cusanovich2018 and SnapATAC performed similarly before and after removing the first PC across all datasets. For Cicero-PCA, removing first PC did not clearly affect its performance in Buenrostro2018 and 10X PBMCs datasets but improved its performance in the down-sampled sci-ATAC mouse dataset.*

*Generally, the methods that implement binarization (e.g. Cusanovich2018, SnapATAC) or that implement cell coverage bias correction (e.g. chromVAR, SnapATAC), tend to be less affected by the sample sequencing depths. Therefore, for these methods we believe that the first PC does not capture the library size and removing it does not help to improve the clustering results. On the contrary, for methods that do not implement any specific step to correct for potential artifacts associated with sequencing depth, the first PC is more likely to capture biologically irrelevant factors and therefore may reduce biology-driven differences. However, this operation must be applied with caution, since removing the first component could also in some cases remove some biological variation (e.g.motif-based chromVAR).”*

Reviewer #2: Chen and colleagues perform an assessment of methods for the processing and analysis of scATACseq data. They use both simulated and real datasets to systematically ascertain which approaches tend to consistently work better in across different settings. Overall, this is a challenging task as there are vast numbers

of ways to do the simulations, fine- tune the parameters for each approach, and evaluate the results. Overall, the authors have been comprehensive and have tackled this task with rigor and transparency in their choices. They have found that several approaches should clearly not be used for de novo clustering and several other algorithms perform relatively well. The ability of snapATAC to rapidly process and analyze large data is also an important finding. Furthermore, they compare the pseudo-bulk vs. clade-bulk strategies. Overall, this benchmarking paper will be of interest to the single cell community and serve as a valuable guide for future methods developers.

Major comments:

1. One analysis that was not performed but would be of interest to researchers in this field is how does sequencing depth affect results. One question always asked is "Will deeper sequencing give new or better-defined clusters?".

Although the effect of noise was assessed, it appears as though the same "depth" was used in all simulations (i.e. 1,000 fragments). What happens if this number is decreased? Do some approaches perform better or worse when the data is sparser?

*We thank the reviewer for the excellent suggestion. To address this point, we have repeated our assessment of performance simulating 5 new datasets with different sequencing depth. As the reviewer was expecting, some methods are more sensitive to the sequencing depth. The results are presented in the main text and Sup Figures 4 and 5.*

*“Using the bulk ATAC-seq bone marrow dataset, we simulated five additional datasets to explore the effect of coverage on clustering performance (5,000 fragments, 2,500 fragments, 1,000 fragments, 500 fragments, 250 fragments respectively per cell).*

*…*

*Next, the effect of the coverage on clustering performance was investigated. We progressively decreased the number of fragments per cell from a high coverage of 5,000 fragments, to a medium coverage of 2,500 fragments and 1,000 fragments, then to a low coverage of 500 fragments and finally to 250 fragments. The performance of all methods declined as coverage was decreased. (Sup Fig. 4b, Sup Fig. 5, Sup Table 4-5-6-7-8). Cusanovich2018, SnapATAC, Scasat, and Control-Naïve are relatively robust to low coverage and outperform other methods. cisTopic worked well with high coverage but in contrast to the above listed methods, was more sensitive to lower coverages (Sup Fig. 5e).”*

*Grouping points 2 and 3 together into a single response below:*

2) Some of the methodology for simulation was unclear. For example, the statement "For a simulated single cell, we generate a vector of per-peak probabilities weighted by the bulk intensity at each peak and the number of fragments in peaks desired, and simulate the observation of a fragment in each peak using the R rbinom function (size = 1)." Could a more formal definition of "weighted by the bulk intensity at each peak and the number of fragments in peaks desired" be given?

3) Similar to above, it was not fully clear in the methods how noise was being added. Could the sentence "To introduce noise, per-peak rates were adjusted by adding a fixed term that was subsequently rescaled" be expanded? Formulas may be helpful as well.

*Thanks for raising these two important points. We acknowledge that the description of our simulation strategy was not clear as also pointed out by Reviewer #1. Therefore, we have significantly expanded the method section to address these concerns. See also response to Reviewer #1 3) for a brief discussion of the simulation rationale.*

4) SnapATAC excludes certain regions that were blacklisted by Encode. Do other algorithms do this at all? Does this step greatly contribute to its increased accuracy or are their other steps that can be attributed to its top rank?

*We thank the reviewer for raising this important point. When the methods were tested on the real datasets, we ran all the methods as they are and followed their complete pipeline, in which only some of the methods excluded the blacklist regions, including cisTopic, Scasat, SnapATAC, and SCRAT. When methods were tested on simulation datasets, since the input is a peaks-by-cells count matrix instead of bam files, we only extracted the core algorithm parts of each method and used the same set of peaks for all methods (without removing the blacklisted regions). Therefore, removing blacklisted regions may only affect the analyses in real datasets.*

*To address the reviewer’s concern and further validate the blacklist contribution to method accuracy, we selected the methods that implemented the step of removing blacklisted regions after pre-processing of the data, including SnapATAC and SCRAT. cisTopic and Scasat remove blacklisted regions prior to the portion of the analysis pipeline covered in our benchmarking framework; therefore, these real datasets were pre-processed using the standard ENCODE pipeline, which does indeed remove blacklisted regions. We compared their performances with and without excluding blacklisted regions and observed that there are no significant differences between them. This data is described in the main text in a new subsection and illustrated in Sup Figure 19.*

*“The effect of excluding regions using the ENCODE blacklist annotation cisTopic, Scasat, SCRAT, and SnapATAC employ a blacklist filtering step to remove features annotated by ENCODE as belonging to a subset of genomic regions, which harbor the potential to produce artifacts in downstream analysis steps [24]. cisTopic and Scasat perform a peak filtering in the pre-processing steps of their pipeline. Our benchmarking pipeline makes use of the ENCODE ATAC-seq pre-processing pipeline, which removes peaks overlapping with regions on the blacklist annotations list. Therefore, we tested the remaining two methods, which do not use peaks as features, SCRAT or SnapATAC. In particular, we wanted to test whether we would observe any change in downstream clustering performance upon opting to perform a blacklist removal step. Through a qualitative and quantitative comparison of clustering performance, we determined that methods, which remove features according to blacklist annotations show no considerable advantage over those that permitted such features (Sup Fig.19).*

Minor comments:

1) In the text the term "naïve" is mostly used for the "control-naïve" dataset. However, in the figures, the term "control" is used more frequently. It would be clearer to use the exact same terminology throughout.

*We thank the reviewer for the nice suggestion. We have replaced all "naïve" with "Control- Naïve"*

2) For the speed performance comparison, were all methods run in parallel with the full 44 cores? This would make a large difference in performance if some methods were parallelizable and others were not.

*We thank the reviewer for raising this important point. We realized that different methods were not using all the available cores even if they were parallelizable, therefore our previous comparison was not fair. Therefore, we re-ran all the methods using exactly 10 cores, since we reasoned that a typical lab may not have access to a machine with 44 cores, and this number of cores better reflects average running time real scenarios where a midsize computing node is used to perform these analyses. For the methods that do not support parallel computing, we are reporting their running time on one core. We believe the updated times should better reflect the relative performance of the evaluated methods.*

*“It is worthwhile to mention that not all the methods of this benchmark support parallel computing. For the methods that support parallel computing, including SnapATAC, chromVAR, and cisTopic, the execution time was reported using 10 cores. For the rest of methods, we run them using a single core. We selected this number reasoning that a typical lab may not have access to a machine with 44 cores and instead may use a mid-size computing node with 8-12 cores.”*

3) Some casual language is used throughout including contractions at lines 293, 319, and 381 ("didn't" and "don't").

*We appreciate the reviewer’s comment. We have removed the casual languages.*

4) This is a grammar mistake in Supp Fig 1 legend. The word "of" is repeated.

*We appreciate the reviewer’s comment. We have fixed the grammar mistake.*

5) For the organization of the "noise" related supplementary figures, it would be easier for the reader to digest if all results/umaps for different noises and clustering algorithms were put in a single plot and then have separate figures for each pipeline/approach (i.e. a single figure containing all of snapATAC noise results). Then one can more readily ascertain the effect of noise on each approach. Currently, it is hard to see the effect of noise on a single approach when having to flip back and forth between figures.

*We thank the reviewer for the suggestion. To better summarize the effect of the noise (and also of the sequencing depth as proposed in point 3) we have generated new summary plots presented in Sup Figure 4.*

**Second round of review**

**Reviewer 2**

The authors have sufficiently addressed my concerns and improved the manuscript by clarifying methods, adding more simulations, and expanding on conclusions. This work greatly adds to the field by evaluating current algorithms and will provide a solid resource for future methods development.

**Reviewer 3**

I really appreciate the efforts that the authors have put in the revision. There are several major improvements from the 1st version and most of the criticize from review #1 have been addressed. Firstly, to ensure the reproducibility of the simulation and clarify the procedure by which it was performed, the authors have provided all scripts used to generate synthetic datasets and all scripts needed to reproduce the entire benchmark analysis. Secondly, to assess the robustness of the different methods and their ability to distinguish cell populations despite sub-optimal sequencing depth, they have generated 5 new datasets with various read coverages in addition to the five synthetic datasets with different noise levels. Thirdly, the authors have discussed “end user” and “developer “perspective separately. 
 However, for the conclusion part, it still need a punch line. The authors conclude that “SnapATAC, Cusanovich2018 , and cisTopic outperform other methods in separating cell populations of different coverages and noise levels in both synthetic and real datasets”. The authors also mentioned that they observed that SnapATAC, cisTopic, Cusanovich2018, and Scasat have even better clustering solutions in their benchmarking framework compared to using their own clustering approach. Since the authors have already add a “Control-Naïve”approach as control method, can they also add a “Maybe best”solution which is a combination of current methods but may outperform all of them? I believe including the “Maybe best” solution can make the conclusion much stronger and demonstrate the value of the benchmark analysis. On the other hand, I think it not worth to publish another paper to provide the “Maybe best” approach.
Other suggestions:
1.     In line 78-81, for the description of “The low copy number results in an inherent per-cell data sparsity, where only 1-10% of expected accessible peaks are detected in single cells from scATAC-seq data, compared to 20-50% of expressed genes detected in single cells from scRNA-seq data.”, where does the number, 1-10%, and 20%-50%, come from? Similar description has also been mentioned in line 27-29. If those number are from other’s review or research article, please provide the reference. If they are from the author’s own result, please provide the data and calculation process. This question has been raised in the first round of review, however, for some reason, the author didn’t address it. 
2.     The effect of blacklists is not comprehensively evaluated. Does the effect have difference in single-cell (i.e. Fluidigm C1 array) and single-nuclei (i.e. 10X genomics) based ATAC assay? There is maybe more intuitive way to help to make conclusion. For example, how many reads come from blacklists region in each real single-cell ATAC dataset? Does signal from blacklists contribute the overall variation?
3.     The authors have already explained more about how to add noise in the simulated data. However, the authors need to make the description easier for non-computation people to understand. For example, what does this noise try to mimic? The non-specific cutting from Tn5, or the complexity of the cell population, or the contamination from another cell type? And in real dataset, what is the range of this noise level?

**Authors Response**

**Point-by-point responses to the reviewers’ comments:**

Reviewer #3: I really appreciate the efforts that the authors have put in the revision. There are several major improvements from the 1st version and most of the criticize from review #1 have been addressed. Firstly, to ensure the reproducibility of the simulation and clarify the procedure by which it was performed, the authors have provided all scripts used to generate synthetic datasets and all scripts needed to reproduce the entire benchmark analysis. Secondly, to assess the robustness of the different methods and their ability to distinguish cell populations despite sub-optimal sequencing depth, they have generated 5 new datasets with various read coverages in addition to the five synthetic datasets with different noise levels. Thirdly, the authors have discussed “end user” and “developer “perspective separately.

However, for the conclusion part, it still need a punch line. The authors conclude that “SnapATAC, Cusanovich2018, and cisTopic outperform other methods in separating cell populations of different coverages and noise levels in both synthetic and real datasets”. The authors also mentioned that they observed that SnapATAC, cisTopic, Cusanovich2018, and Scasat have even better clustering solutions in their benchmarking framework compared to using their own clustering approach.

Since the authors have already add a “Control-Naïve”approach as control method, can they also add a “Maybe best”solution which is a combination of current methods but may outperform all of them? I believe including the “Maybe best” solution can make the conclusion much stronger and demonstrate the value of the benchmark analysis. On the other hand, I think it not worth to publish another paper to provide the “Maybe best” approach.

*We appreciate and agree with the insightful comments regarding a presentation of a method yet to be developed that is ideal. Indeed, based on the analyses presented, a method that combined the modules presented in Fig. 2 may outperform currently available methods. However, in order to make an educated approach to answering this question, we would need to evaluate all of the most promising combinations of modules that might lead to a ‘maybe best’ solution method; this would require a significant amount of work that we believe is outside the scope of this benchmark paper.*

*We do still believe the suggested idea to be an important point. To emphasize this and our agreement with the sentiment of the reviewer’s comments, we have expanded the conclusion as follows:*

*Based on the presented analyses, we showed that a few methods outperformed others in terms of clustering performances and running time. However, we believe that a method based on the combination of modules used in the best methods may outperform them. In support of this idea, we found that simply altering the normalization step or clustering approach significantly improved the downstream clustering performance of some methods. With this in mind, we believe it may be possible to build a ‘maybe best’ scATAC-seq computational method by adopting the most impactful modules from the best-performing methods evaluated. Such modules include: (1) defining features as peaks/fixed-size bins, (2) count peaks/bins + binarization/TF-IDF/coverage bias correction, (3) dimensionality reduction, (4) community detection-based clustering. However, in exploring the best combination of modules to build such a method, it is important to consider the balance between clustering performance, running time, and memory requirements. Such practical considerations are key to enabling the analysis of large datasets.*

Other suggestions:

1. In line 78-81, for the description of “The low copy number results in an inherent per-cell data sparsity, where only 1-10% of expected accessible peaks are detected in single cells from scATAC-seq data, compared to 20-50% of expressed genes detected in single cells from scRNA-seq data.”, where does the number, 1-10%, and 20%-50%, come from? Similar description has also been mentioned in line 27-29. If those number are from other’s review or research article, please provide the reference. If they are from the author’s own result, please provide the data and calculation process. This question has been raised in the first round of review, however, for some reason, the author didn’t address it.

*We apologize if we didn’t fully address this point in the past revision. For scRNA-seq, this range is now slightly adjusted to 10%~45% based on two recent benchmark papers assessing different scRNA-seq protocols ( Mereu et al. 2019 and Ding et al. 2019 [1, 2]) . For scATAC-seq, we estimated this range based on the datasets analyzed in this manuscript. Briefly, we first calculated the number of detected peaks for each single cell (a peak is defined as detected if it is covered by at least one read in a given single cell). Then we divided the number of detected peaks in each single cell by the total number of peaks that can be detected in a bulk experiment or pseudo-bulk track.*

*We have updated the manuscript by adding the missing references and a new supplementary figure (Supplementary Figure 1) to support our claim.*

2. The effect of blacklists is not comprehensively evaluated. Does the effect have difference in single-cell (i.e. Fluidigm C1 array) and single-nuclei (i.e. 10X genomics) based ATAC assay? There is maybe more intuitive way to help to make conclusion. For example, how many reads come from blacklists region in each real single-cell ATAC dataset? Does signal from blacklists contribute the overall variation?

*We thank reviewer for raising this important point. We expanded significantly this part with the following new analyses.*

*First, as suggested, we estimated how many reads/bins overlap the blacklisted regions. Blacklisted regions are those features annotated by ENCODE as belonging to a subset of genomic regions, which harbor the potential to produce artifacts in downstream analyses. In particular, we calculated for all the three technologies: 1) the proportion of reads mapped into blacklist regions to the total number of reads; 2) the proportion of bins with at least one read overlapping the blacklisted regions to the total number of bins with at least one read (since this better corresponds to the feature set used by several of the evaluated methods)*

*We observe that for 10X genomics and sciATAC-seq the % reads mappable to blacklist regions is only ~1%, while for the Fluidigm C1 based assay used in Buenrostro2018 is much higher i.e. ~50%. However, when considering bins, only ~0.01-0.02%.of bins with at least one read correspond to blacklist regions for all the three technologies. Given this negligible proportion, we believe that the signal from blacklists can hardly contribute to the overall variation.*

*Second, to test this in a more quantitative way, we expand our previous analysis to test the effect of the blacklist regions on clustering performance, initially performed only on the Buenrostro2018 dataset, to the 10X PBMCs and downsampled sciATAC-seq datasets. As expected, based on the previous considerations, we didn’t observe a noticeable effect on the downstream analysis suggesting that blacklist regions signal contribute minimally to the overall variation and population separability.*

*We have expanded the section “The effect of excluding regions using the ENCODE blacklist annotation” with the following text:*

*Blacklisted regions are those features annotated by ENCODE as belonging to a subset of genomic regions, which harbor the potential to produce artifacts in downstream analyses. In order to assess the potential contribution that blacklist regions could have on the overall variation and population separability, we calculated: 1) The proportions of reads mapped to blacklisted regions and 2) the proportions of bins with at least one read overlapping a blacklisted region vs the proportion of bins containing reads that do not overlap blacklist regions. Such a ratio corresponds closely to the feature set used by several of the evaluated methods*

*We observed that for 10X genomics and sciATAC-seq the % reads mappable to blacklisted regions is only ~1%, while for the Fluidigm C1 based assay used in Buenrostro2018 is much higher, ~50%. However, when considering bins, only ~0.01-0.02%.of bins with at least one read correspond to blacklist regions for all three technologies (Additional file 1: Fig. S20). This fraction of bins containing one or more reads in a blacklisted region is likely negligible and we hypothesize that the variation in the signal from reads in blacklisted regions is similarly negligible.*

*It is worth noting that cisTopic, Scasat, SCRAT, and SnapATAC employ a blacklist filtering step to remove features annotated by ENCODE as belonging to a subset of genomic regions, which harbor the potential to produce artifacts in downstream analysis steps [3]. cisTopic and Scasat perform a peak filtering in the pre-processing steps of their pipeline. Our benchmarking pipeline makes use of the ENCODE ATAC-seq pre-processing pipeline, which removes peaks overlapping with regions on the blacklist annotations list.*

*SCRAT and SnapATAC do not use peaks as features, therefore they are the only methods potentially affected by blacklist-mapped artifacts. We tested whether we would observe any change in downstream clustering performance upon opting to perform a blacklist removal step. Through a qualitative and quantitative comparison of clustering performance across the datasets generated by the three different technologies (10X genomics, sciATAC and Fluidigm C 1), we determined that methods, which remove features according to blacklist annotations show no considerable advantage over those that permitted such features (Additional file 1: Fig. S21).*

3.The authors have already explained more about how to add noise in the simulated data. However, the authors need to make the description easier for non-computation people to understand. For example, what does this noise try to mimic? The non-specific cutting from Tn5, or the complexity of the cell population, or the contamination from another cell type? And in real dataset, what is the range of this noise level?

*Thank you for raising these important points. The noise level tries to mimic the non-specific cutting from Tn5, in fact in our simulations based on equation 2 section “Simulated scATAC-seq datasets” we use this parameter to set the balance between two distributions: the first considers the population structure based on the probability to sample from different peaks that can be more or less cell type specific, while the second is an uninformative, uniform distribution.*

*Estimating this noise level from real data is challenging since at any given region of the genome we could observe reads coming from specific and non-specific cutting. However, if we assume that reads in regions defined as peaks by bulk or pseudobulk will be dominated by specific cutting and that regions outside peaks will be dominated by non-specific cutting we can get a reasonable approximation comparing their rates i.e. considering the number of reads falling outside peaks over the total number of reads (but excluding the blacklist regions). Using this approach, we estimated the following noise levels: 0.38 for the Buenrostro2018, 0.22 for the 10X PBMC data and 0.62 for the sci-ATAC-seq mouse data.*

*We have modified the manuscript to include a short paragraph to discuss these important points.*

*The noise level we simulated attempts to mimic the non-specific cutting from Tn5. To give a sense of the range of this parameter on real data, we considered simply the number of reads falling outside peaks over the total number of reads (excluding blacklisted regions). This calculation assumes that reads in regions defined as peaks by a bulk or pseudobulk measurement will be dominated by specific cutting and that regions outside peaks will be dominated by non-specific cutting. Using this approach, we estimated the following noise levels: 0.38 for the Buenrostro2018 dataset, 0.22 for the 10X PBMC dataset and 0.62 for the sci-ATAC-seq mouse dataset. We would like to point out that these rates may be slightly underestimated; a more careful estimation would require one to consider the fact that, at any given region of the genome, reads could be observed from specific and non-specific cutting.*

*References*

*1.	Mereu, E., et al., Benchmarking Single-Cell RNA Sequencing Protocols for Cell Atlas Projects. BioRxiv, 2019.*

*2.	Ding, J., et al., Systematic comparative analysis of single cell RNA-sequencing methods. 2019.*

*3.	Amemiya, H.M., A. Kundaje, and A.P. Boyle, The ENCODE Blacklist: Identification of Problematic Regions of the Genome. Sci Rep, 2019. 9(1): p. 9354.*
